# Metagenomic Thermometer

**DOI:** 10.1093/dnares/dsad024

**Published:** 2023-11-06

**Authors:** Masaomi Kurokawa, Koichi Higashi, Keisuke Yoshida, Tomohiko Sato, Shigenori Maruyama, Hiroshi Mori, Ken Kurokawa

**Affiliations:** Genome Evolution Laboratory, National Institute of Genetics, 1111 Yata, Mishima, Shizuoka 411-8540, Japan; Genome Evolution Laboratory, National Institute of Genetics, 1111 Yata, Mishima, Shizuoka 411-8540, Japan; Department of Biological Information, Tokyo Institute of Technology, 2-12-1 Ookayama, Meguro-ku, Tokyo 152-8550, Japan; Department of Biological Information, Tokyo Institute of Technology, 2-12-1 Ookayama, Meguro-ku, Tokyo 152-8550, Japan; Earth-Life Science Institute, Tokyo Institute of Technology, 2-12-1 Ookayama, Meguro-ku, Tokyo 152-8551, Japan; Earth-Life Science Institute, Tokyo Institute of Technology, 2-12-1 Ookayama, Meguro-ku, Tokyo 152-8551, Japan; Genome Evolution Laboratory, National Institute of Genetics, 1111 Yata, Mishima, Shizuoka 411-8540, Japan; Genome Diversity Laboratory, National Institute of Genetics, 1111 Yata, Mishima, Shizuoka 411-8540, Japan; Department of Biological Information, Tokyo Institute of Technology, 2-12-1 Ookayama, Meguro-ku, Tokyo 152-8550, Japan; Genome Evolution Laboratory, National Institute of Genetics, 1111 Yata, Mishima, Shizuoka 411-8540, Japan; Department of Biological Information, Tokyo Institute of Technology, 2-12-1 Ookayama, Meguro-ku, Tokyo 152-8550, Japan; Earth-Life Science Institute, Tokyo Institute of Technology, 2-12-1 Ookayama, Meguro-ku, Tokyo 152-8551, Japan

**Keywords:** metagenome, temperature, hot spring, human gut, community assembly

## Abstract

Various microorganisms exist in environments, and each of them has its optimal growth temperature (OGT). The relationship between genomic information and OGT of each species has long been studied, and one such study revealed that OGT of prokaryotes can be accurately predicted based on the fraction of seven amino acids (IVYWREL) among all encoded amino-acid sequences in its genome. Extending this discovery, we developed a ‘Metagenomic Thermometer’ as a means of predicting environmental temperature based on metagenomic sequences. Temperature prediction of diverse environments using publicly available metagenomic data revealed that the Metagenomic Thermometer can predict environmental temperatures with small temperature changes and little influx of microorganisms from other environments. The accuracy of the Metagenomic Thermometer was also confirmed by a demonstration experiment using an artificial hot water canal. The Metagenomic Thermometer was also applied to human gut metagenomic samples, yielding a reasonably accurate value for human body temperature. The result further suggests that deep body temperature determines the dominant lineage of the gut community. Metagenomic Thermometer provides a new insight into temperature-driven community assembly based on amino-acid composition rather than microbial taxa.

## 1. Introduction

The effect of temperature on microbial growth has long been a topic of research.^1^ It is generally accepted that each microbial species has an optimal growth temperature (OGT), and that growth rates decrease as the temperature deviates from OGT.^2,3^ This may be attributable to the temperature sensitivity of the structures of their biomolecules.^4^ Therefore, a number of relationships have been discovered between OGT and genomic information. For example, the GC content of microbial tRNAs and rRNAs correlates with OGT.^5,6^ Also, compared with mesophiles, thermophiles have a higher proportion of purine bases (A and G) in their mRNAs.^7,8^ Moreover, the amino-acid composition of microbial proteins is also closely correlated with OGT. For example, the overall proportion of charged amino acids is relatively high in thermophiles.^9,10^ Interestingly, Zeldovich et al. found that the fraction of seven amino acids, namely Ile, Val, Tyr, Trp, Arg, Glu, and Leu (abbreviated as *F*_*IVYWREL*_), in a proteome strongly correlated with OGT; and indeed OGT could be accurately predicted by a simple equation with *F*_*IVYWREL*_ as the only variable.^11^

The OGT of a microorganism also reflects the temperature of its natural habitat. Thermophiles, which by definition have an OGT of ≥45°C,^12^ are usually found in hot springs. For example, species of the phylum Chloroflexi^13–17^ are often the predominant species found in hot springs near 50°C, and Aquificae^13,15,16,18,19^ and Deinococcus-Thermus^16,18,20^ include the major species in hot springs warmer than 50°C. Therefore, we hypothesized that environmental temperature could be predicted based on the overall *F*_*IVYWREL*_ value for all microorganisms present in a particular habitat.

Here, we report a ‘Metagenomic Thermometer’ as a means of predicting environmental temperature based on *F*_*IVYWREL*_ obtained for amino-acid sequences translated from metagenomic DNA sequences. First, we applied Metagenomic Thermometer for metagenomic data from public databases to clarify the applicability and limitations. Next, we conducted a demonstration experiment to confirm the accuracy of the Metagenomic Thermometer by constructing an artificial hot water canal. Finally, we tested whether the Metagenomic Thermometer could be applied to the human gut metagenomic data. Overall, the results demonstrate that the Metagenomic Thermometer is effective as a method for predicting temperature and provides important insights for understanding the contribution of temperature to the composition of microbial communities.

## 2. Materials and methods

### 2.1. Acquisition of public sequence data

A total of 33 hot spring, 616 human gut, 14 soil, 36 stream, 16 sewage, 40 plant water, and 348 ocean metagenomic samples were used for analyses. All public metagenomic datasets were downloaded from the NCBI Sequence Read Archive (SRA) database using the SRA Toolkit. Accession numbers and metadata for sequence data are listed in Supplementary Tables S1–S4. For hot springs, shotgun metagenomic data annotated with hot springs metagenome (taxid: 433727) and temperature information were searched in the SRA database. Available metagenomic data for hot springs found in the literature were also added. As of May 2021, the NCBI Sequence Read Archive database contained shotgun metagenomic sequences for 39 hot springs with temperature data. We removed samples with a fastq file size of <10 MB or if contamination of the biological sample was suspected, leaving 33 samples for analysis. Eight metagenomic data from hot water canal obtained in this study were also used for the analysis. Metagenomic data for soil,^21,22^ stream and wastewater,^23^ ocean,^24^ and human gut^25^ were obtained from the studies listed in the references.

### 2.2. Construction of a hot water canal

A hot water canal was constructed in February 2014 to enable experimentation at different water temperatures. Water for the canal was drawn from Kin-yu hot spring, which is located in the Kirishima area in Kagoshima Prefecture in Japan. The Kirishima area is located on the volcanic front where the Philippine Sea plate sinks into the Eurasian plate,^26^ and ~20 volcanoes exist in the area of ~20 km × 30 km.^27^ Thus, this region has numerous hot springs that range in temperature from ~40°C to 97°C and in pH from ~2 to 9.^28–30^ Kin-yu hot spring has a temperature range of 70–85°C with near-neutral pH.^28,30^

The canal length was ~20 m and was constructed beside the Kin-yu hot spring source using U-shaped gutters. Water was drawn from the source through a pipe, and the downstream outlet was naturally open. A net was placed over the canal to prevent clogging by leaves, yet soil and microbes could enter freely.

### 2.3. Sample collection

All samples from the canal were collected in February 2016. Surface-sediment samples (~15 g) from the canal were placed into 50-ml conical tubes using sterilized steel spatulas. To avoid contamination between downstream and upstream sediment, sampling was carried out from downstream to upstream. Also, different spatulas were used for each sampling. Collected samples were immediately placed in a cooler and transported to the laboratory, where they were stored at –20°C. Immediately before sampling, the pH and temperature of the canal water were measured using a portable conductivity/pH meter (WM-32EP, DKK-TOA Co.), and oxidation–reduction potential and dissolved oxygen of the water were measured using a portable dissolved oxygen/pH meter (DM-32P, DKK-TOA Co.) at a site just above each sampling point. Sampling was carried out in three replicates at each point. One was for shotgun sequencing, another was for amplicon sequencing, and the other was as a spare.

### 2.4. DNA extraction

Frozen sediment samples were thawed at room temperature, and DNA was extracted from the samples using the PowerSoil DNA Isolation kit (MO BIO; version 01152013) with a Micro SmashMS-100R (TOMY SEIKO) for bead homogenization (3,000 rpm, 30 s). Each sample of extracted DNA was in a volume of 50 µl. DNA concentration was initially approximated using a Qubit 2.0 Fluorometer (Thermo Fisher Scientific), with subsequent accurate measurement using a Model 2100 Bioanalyzer (Agilent Technologies).

### 2.5. PCR amplification of the 16S rRNA gene

The V3–V4 region of the 16S rRNA gene was amplified from the extracted DNA using universal primers 342F and 806R.^31^ PCR mixtures contained 21 µl sterilized distilled water, 3 µl template DNA solution, 25 µl Premix Taq Hot Start Version (TaKaRa Bio), 0.5 µl forward primer, and 0.5 µl reverse primer. The thermal cycler protocol was 95°C for 2 min, followed by 30 cycles of 98°C for 10 s, 50°C for 30 s, and 72°C for 40 s, with final extension at 72°C for 10 min. PCR products were separated by electrophoresis (1.5% agarose gel) and purified using the Wizard SV Gel and PCR Clean-Up System (Promega). Purified DNA was quantified using the Model 2100 Bioanalyzer with a High-Sensitivity DNA kit (Agilent Technologies; version 01.02).

### 2.6. Sample preparation for shotgun metagenomic sequencing

Extracted DNA samples were processed using the Nextera DNA Library Preparation kit (Illumina) according to the Nextera DNA Sample Preparation Guide #15027987 and purified using the DNA Clean & Concentrator™ (ZYMO RESEARCH). Finally, libraries were subjected to quantitative PCR using the KAPA Library Quantification kit (NIPPON Genetics Co., Ltd.) according to the manufacturer’s protocol for Illumina (2013.9.6 rev6).

### 2.7. DNA sequencing

Paired-end DNA sequencing was performed by the Earth-Life Science Institute (Tokyo Institute of Technology) using MiSeq (Illumina) according to the MiSeq System Quick Reference Guide For MiSeq Software 2.3. In the 16S rRNA amplicon sequencing, PhiX was spiked into the DNA samples at 50% relative concentration for quality control. Metagenomic sequencing reads were deposited in the DNA Data Bank of Japan with accession number DRA012733.

### 2.8. Quality control of sequence reads

Adapter trimming and removal of low-quality reads were performed using fastp, v0.20.0.^32^ The lower limit of read length was set to 50 bp, and the allowable limit of the number of ambiguous nucleotides (N) per read was set to 1. The statistics of the sequence data were examined using SeqKit, v0.16.1,^33^ and are summarized in Supplementary Tables S5 and 6.

### 2.9. Prediction of OGT

The OGT for each bacterial species was predicted based on the method reported by Zeldovich et al.^11^ Amino-acid sequence fasta files for coding regions of focal bacterial species were downloaded from the NCBI Genome database. *F*_*IVYWREL*_ was calculated for all coding sequences of focal species, and then OGT for each species was predicted by applying the following formula:


$$\begin{array}{l} \begin{array}{l} OGT=937\times F_{IVYWREL}\;-335\; \end{array} \end{array}$$ (1)

### 2.10. Calculation of the metagenomic predicted temperature and implementation of web application

Quality-filtered metagenomic DNA fastq files were converted to DNA fasta files using SeqKit, v0.16.1. Open reading frames were identified from the DNA fasta files using Prodigal, v2.6.3,^34^ resulting in the corresponding amino-acid fasta files. *F*_*IVYWREL*_ values were calculated from the amino-acid fasta files, and Eq. 1 was applied to calculate metagenomic predicted temperature (MPT) instead of calculating OGT. Hereafter, we call this temperature prediction method the Metagenomic Thermometer, for which a web application (written in Python) is freely available at http://metathermo.jp/. Users can upload a fasta or fastq file for shotgun sequencing data and receive a corresponding MPT value.

### 2.11. Acquisition of published information for OGT

The OGT for each bacterial species based on actual measurements was obtained according to the online dataset: https://doi.org/10.5281/zenodo.1175608. This dataset was summarized by Engqvist and published in 2018.^35^ This dataset was constructed by integrating information from six major databases and contains OGT data for 21,498 non-redundant microbes.

### 2.12. Taxonomic profiling of microbial communities

The taxonomic composition of each metagenomic sample was determined using VITCOMIC2^36,37^ based on metagenomic DNA data in the fasta files (forward read files only). VITCOMIC2 identifies 16S rRNA gene sequences from the input data and returns results for both genus- and phylum-level taxonomic composition. The abundance of each taxon in each sample was converted to a relative value for each sample and used for subsequent analysis. The Bray–Curtis dissimilarity between each microbial community was calculated using the R^38^ package vegan.^39^

### 2.13. LEA mapping

To analyse the characteristics of the microbial community composition, metagenomic DNA sequence data in fasta from 616 human gut metagenomic samples were processed using VITCOMIC2. The resulting files were then uploaded to the LEA web application (http://leamicrobe.jp),^40^ to obtain coordinate information of those samples within the LEA space. Subsequently, the LEA’s REST API (http://snail.nig.ac.jp/leaapi) was utilized to retrieve the coordinates and metadata of the LEA reference samples and topics. This information was used to represent the microbiome sample distribution within the LEA reference space in the form of a scatter plot. The plotted samples were coloured based on the gender information provided by the reference paper.^25^ A gradient colour was visualized in accordance with metagenomic predicted temperatures calculated by our method.

### 2.14. Removal of sequences derived from human gut microorganisms

The metagenomic sequences from Stream, plant water, and sewage were mapped to the UHGG (Unified Human Gastrointestinal Genome), v2.0,^41^ using the BWA-MEM, v0.7.17,^42^ with default options. Subsequently, the samtools, v1.9, ^43^ view command was utilized with the −f 4 option to extract only the unmapped reads, which were then employed for temperature prediction.

### 2.15. Domain classification of metagenomic sequences

Domain classification of metagenomic sequences was conducted using Kraken2, v2.1.2,^44^ with default options. Quality-filtered fastq files (forward read files only) for metagenomic sequences were queried against Kraken PlusPFP database. Based on the report file, the proportion of domains was calculated within domain-annotated sequences.

## 3. Results

### 3.1. Evaluation of applicability of the Metagenomic Thermometer using public metagenomic data

**Figure 1.**
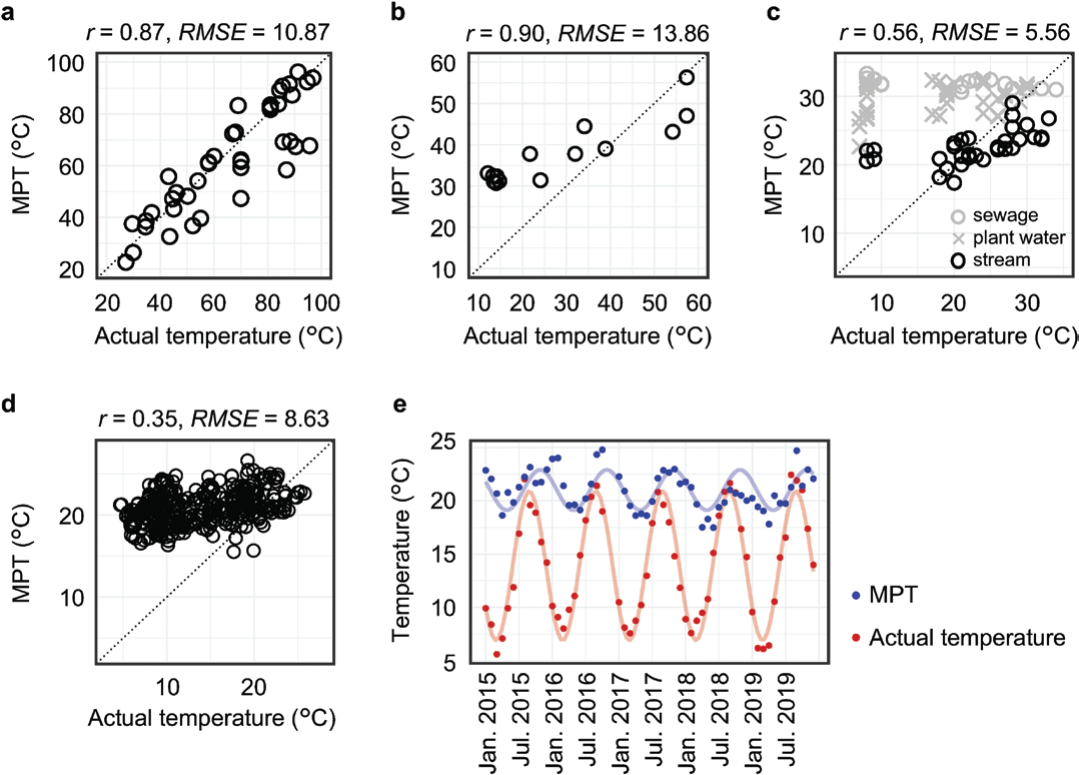
Temperature prediction for public metagenomic data. Temperature prediction of metagenomic samples of hot spring (a), soil (b), wastewater and stream (c), and ocean (d). Each point is one metagenomic sequences. The dotted line represents the perfect match between the actual and MPT. Pearson’s correlation coefficient and root mean squared error are shown above each graph. In graph (c), values are shown for the result of stream. (e) Periodic variation of water temperature and MPT. Each plot is the average of six measurements in each time point. Regression lines for sine wave are shown. The formulas of line are *y* = 1.9 × sin 2π(*x* + 4.2)/12 + 21 in MPT, and *y* = 7 × sin 2π(*x* + 6.1)/12 + 13.9 in water temperature, respectively.

To evaluate the ability of the Metagenomic Thermometer to predict environmental temperature, we applied Metagenomic Thermometer to publicly available metagenomic shotgun sequencing data for hot springs, soils, wastewaters and streams, and oceans. Metagenomic Thermometer could well predict the temperature of hot spring (Fig. 1a). In the case of soil, stream, and ocean, MPT is significantly correlated with measured temperature (*P* < 0.01), but the temperature tends to be slightly higher under low-temperature conditions (Fig. 1b–d). Figure 1c shows the results for a dataset of a system where urban sewage is treated in a plant and discharged into a natural stream.^23^ In contrast to stream, sewage and plant water samples tend to result in higher MPTs. This is thought to be due to contamination of sewage by human-associated microbes whose OGT is higher than the sewage temperature. To validate this hypothesis, we mapped metagenomic sequences to the UHGG and found that a greater number of sequences were mapped to the UHGG in sewage and plant water compared to stream (Supplementary Fig. S1a). Removing the reads mapped to UHGG actually reduced MPT in plant and sewage compared with stream (Supplementary Fig. S1b). Furthermore, it was confirmed that removing reads mapped to the UHGG led to a partial improvement in accuracy of temperature prediction in sewage and plant water (Supplementary Fig. S1c). Investigation of time-series changes in MPT of ocean samples revealed year-round cycles (Fig. 1e). Moreover, the cycle of MPT was delayed by about 2 months compared with that of water temperature. This suggests that it takes a long time for microbial communities to respond to temperature changes. In summary, the Metagenomic Thermometer would be able to predict the temperature of environments that are warm, have little temperature change, and have little influx of microorganisms from the external environment. The high prediction accuracy of hot spring temperature is probably attributed to the warm and stable temperature of hot springs.^45^

### 3.2. Demonstration experiment using artificial hot water canal

**Figure 2.**
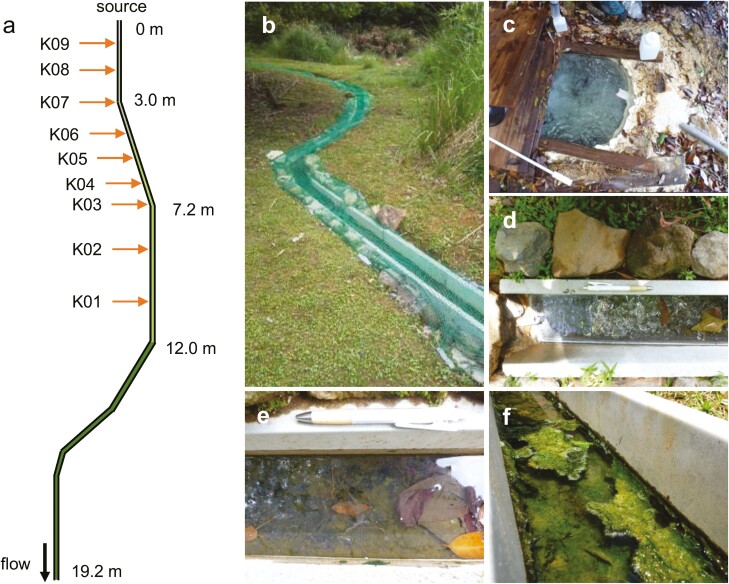
Schematic diagram of a hot water canal. (a) The elongated line represents the hot water canal. Water flow was from top to bottom, and the distance from the starting point of the flow is shown above the line. Sediment colour was yellow or grey up to ~1 m, dark grey or dark green up to ~4 m, light green up to ~12 m, and light green downstream from there. Orange arrows indicate the sampling points. (b) Photograph of the canal viewed from a downstream point. (c) The source of the Kin-yu. The pipe leads to the canal. (d) Upstream of canal connected to the source by a pipe. (e) Sediment with a light-green biomat. (f) Sediment with a thick, light-green biomat.

To more precisely evaluate the accuracy of the Metagenomic Thermometer, we constructed an artificial hot water canal as an environment in which conditions other than temperature could be held constant (Fig. 2). Table 1 lists the physicochemical conditions at nine sampling points (K-01 to K-09, downstream to upstream) in the experimental hot water canal. As expected, the temperature dropped from upstream to downstream (from 60.0°C to 29.6°C). The pH range was 7.62–8.31 (weakly alkaline) over all sampling points.

**Table 1.** Physicochemical properties of samples

Sampling point	Temperature (°C)	pH	ORP (mV)	DO (mg/l)
K-01	29.6	8.00	–19	0.97
K-02	34.7	7.99	–19	0.79
K-03	36.8	8.01	–54	0.87
K-04	40.0	8.31	–86	0.84
K-05	43.1	8.24	–105	0.58
K-06	44.6	8.28	–104	0.56
K-07	50.2	8.28	–134	0.57
K-08	54.0	7.91	–180	0.34
K-09	60.0	7.62	–272	0.49

DO: dissolved oxygen; ORP: oxidation–reduction potential.

**Figure 3.**
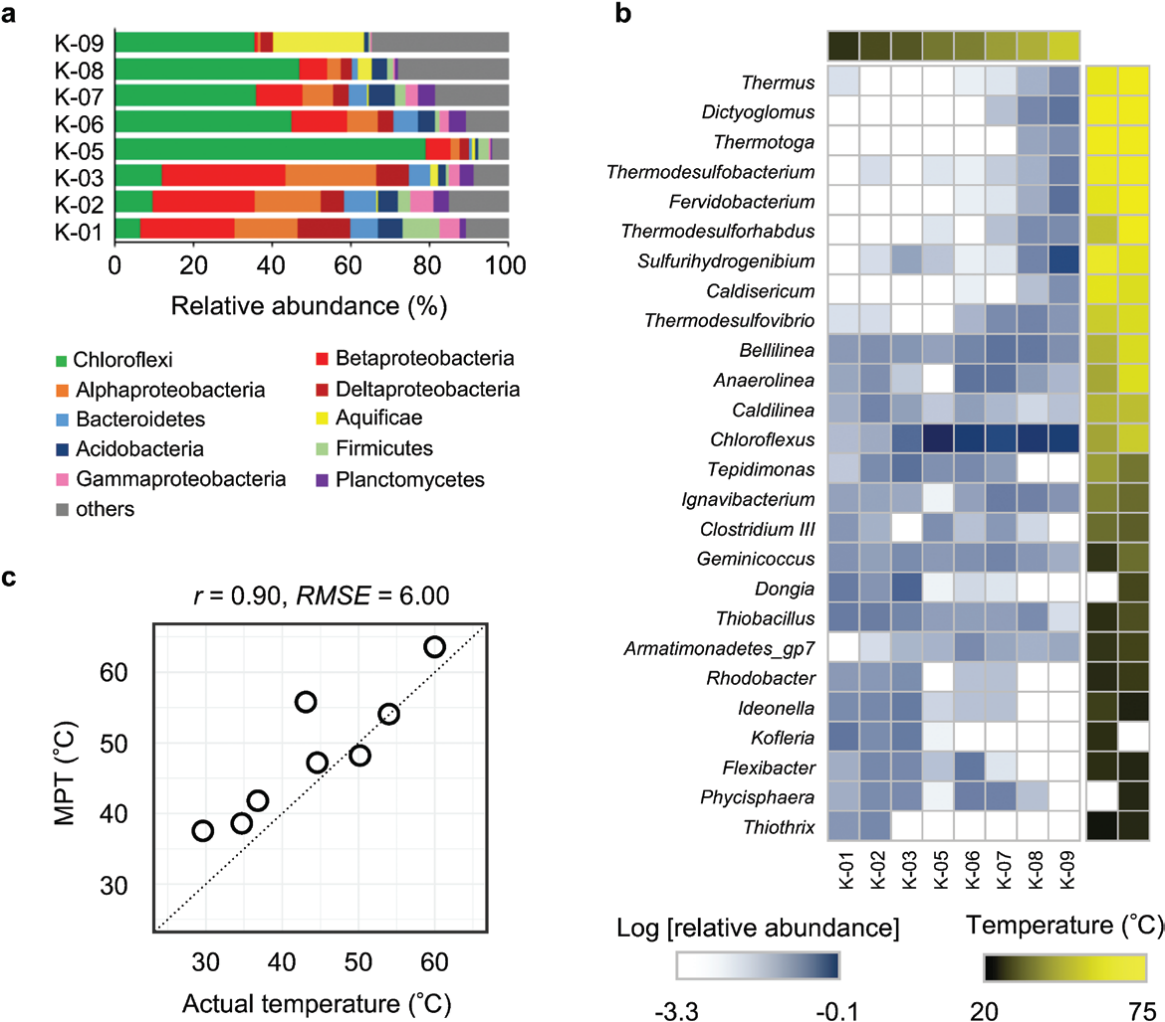
Analysis of the microbial community of the experimental hot water canal. (a) Relative abundance of microbial phyla/classes in each sample. Colours correspond to the phyla or classes (for Proteobacteria) as shown in the legend. Labels on the left represent the sample points of the canal indicated in Fig. 1a. (b) Heatmap of major genera abundance. Genera with relative abundance of more than 2% in any sample are shown. Each column represents a single sampling point, and the colour of the above cells indicates the temperature of the samples corresponding to the scale. The two columns of coloured squares shown on the right show the median OGT for the species included in the genus; the left column shows the OGT obtained based on the dataset (https://doi.org/10.5281/zenodo.1175608), and the right column shows the OGT predicted based on F_IVYWREL_. (c) Plot of MPT versus actual temperature for eight hot water canal sampling points. The dotted line represents the perfect match between the predicted and actual temperatures.

A total of 48,150,715 high-quality shotgun metagenomic reads with average length 181 bp were obtained from eight of the nine hot water canal samples. K-04 is only shown as an amplicon analysis result, because the DNA concentration extracted from sample K-04 was insufficient for shotgun metagenomic sequencing. DNA sequencing statistics are summarized in Supplementary Table S5 and 6. The phylum-level taxonomic composition for each sample was examined using VITCOMIC2 (Fig. 3a). Phylum Cyanobacteria were omitted from the graphs as they are challenging to differentiate from chloroplasts. Each sample had 21–31 microbial phyla, and 34 different phyla were represented in total. Chloroflexi and Proteobacteria were the predominant phyla in the microbiome among all samples, representing ~34% and 33% of the population on average, respectively, followed by two other phyla, Bacteroidetes (4.2%) and Aquificae (3.8%). Note that Proteobacteria was classified at the class level according to the VITCOMIC2 results. The results of the 16S rRNA gene amplicon sequencing, performed on samples from the same hot water canal site for the shotgun analysis, are shown in Supplementary Fig. S2.

To better understand the effect of temperature on microbial community composition, we investigated the relationship between the temperature of the sampling point and OGT of the predominant genus. For genera representing>2% relative abundance in at least one sample, the median OGT of the species belonging to that genus was investigated (Supplementary Table S7). OGT was obtained from the public dataset and also predicted using *F*_*IVYWREL*_ values from genetic information. The results revealed that genera with relatively higher OGT tended to be more abundant in higher-temperature samples, and vice versa (Fig. 3b). Therefore, although OGT is defined by cultures in laboratory, it also seems to reflect relative prosperity of a bacterial species in nature.

We applied the Metagenomic Thermometer to metagenomic sequences of the sediment samples and revealed that the temperature of sampling point could be predicted with an accuracy (Fig. 3c). One exception was sample K-05, for which the predicted temperature was significantly higher than the actual temperature. This may be attributable to the large proportion of Chloroflexi in K-05 compared with other samples (Fig. 3a). The average predicted OGT of species in *Chloroflexus*, the predominant genus of Chloroflexi in K-05, was ~60.2°C. The large proportion of Chloroflexi in K-05 is probably attributable to sample collection bias, such as contamination with biomat debris. Analysis of the 16S rRNA amplicon sequences of sample K-05, which was an independently collected replicate sample from the same sampling point, showed that the relative abundance of Chloroflexi in K-05 was not so high (Supplementary Fig. S2). For reference, the prediction accuracy of sample temperature is improved using seven samples except for K-05 (*r* = 0.97, *RMSE* = 4.25).

### 3.3. Application of the Metagenomic Thermometer for temperature prediction of the human gut

**Figure 4.**
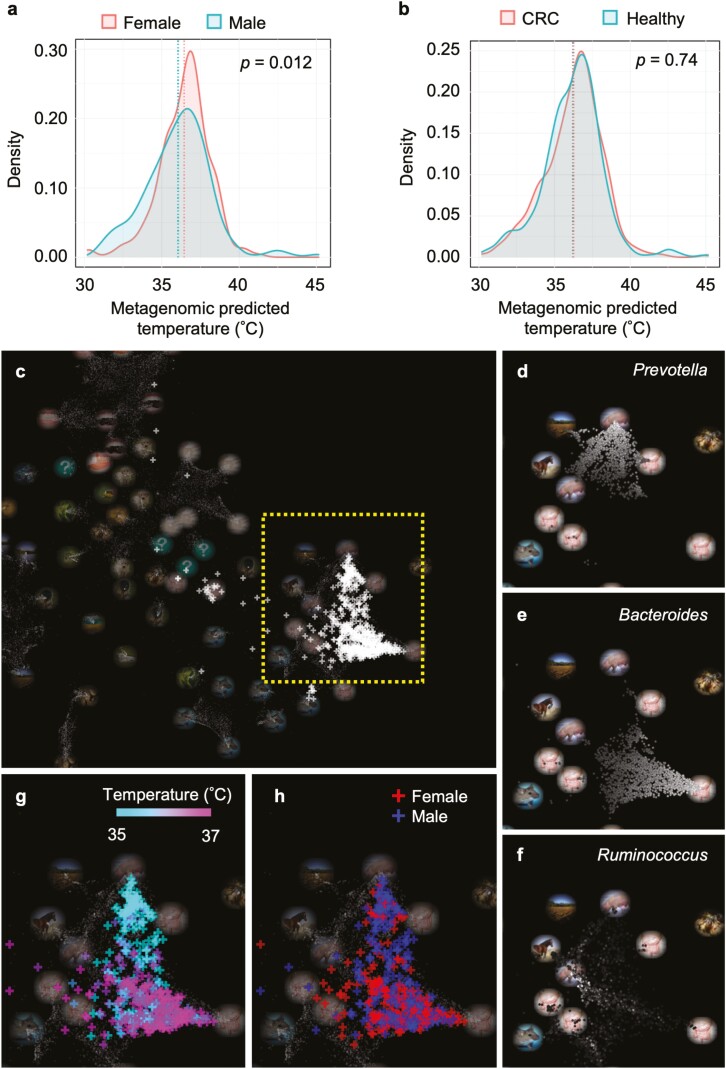
Temperature prediction based on the human gut microbiome. Distributions of MPT calculated based on the gut microbiome is shown separately according to gender (a) and whether individuals had colorectal cancer (CRC) (b). Vertical lines represent mean values and *P*-value of two-sided Welch’s *t*-test are shown in figures. (c) LEA map with all 616 samples (white plus signs). The various circular photographs represent environmental topics, which corresponds to microbial communities that represent the latent environment. Samples or environmental topics that are relatively close in the LEA space have similar community composition. The area enclosed by the yellow dotted rectangle is enlarged in panels (d–h). The myriad points represent metagenomic samples stored in MicrobeDB.jp, and the higher the proportion of *Prevotella* (d), *Bacteroides* (e), and *Ruminococcus* (f) in each sample, the whiter is the sample colour. Samples used in this study are colour coded according to predicted temperature (g) or gender (h).

At last, the Metagenomic Thermometer was applied to human gut microbiome. We obtained the human gut metagenomic data, which consisted of 616 individuals, including healthy subjects and patients with colorectal cancer.^25^ This dataset was suitable for our study owing to the large number of samples and metadata available for individuals. The average MPT value for all samples was 36.22 ± 1.98°C (standard deviation). Given that the average human body temperature range is 36–37°C,^46,47^ the Metagenomic Thermometer was able to predict body temperature with high accuracy. Stratification of the data by gender revealed that the predicted temperature tended to be higher for females (36.45 ± 1.66°C) than males (36.02 ± 2.16°C) (Fig. 4a). Large-scale studies of human body temperature have also shown that female body temperature tends to be higher than that of males.^46,48^ Body temperature has also been reported to correlate with age^46,47^ and body mass index,^46^ but our results indicated no correlation between predicted temperature and these two parameters (Supplementary Fig. S3). As for a comparison between colorectal cancer patients and healthy subjects, there were no significant differences in the distribution of predicted body temperatures (Fig. 4b).

To investigate the relationship between MPT and community composition, all samples were mapped onto the LEA global map. LEA is a web application that visually expresses microbiome similarities between samples and the strength of their relevance to environmental topics.^40^ Most of the samples were placed around the environmental topics representing the gut (Fig. 4c). The human gut microbiome can be classified into three enterotypes based on whether the predominant genus is *Prevotella*, *Bacteroides*, or *Ruminococcus*.^49^ Visualizing the abundance of these three genera in the public data contained in the LEA global map revealed that the three enterotypes could be clustered in different areas on the LEA global map (Fig. 4d–f). Visualization of the MPT of samples by colour scale revealed that samples with a relatively low MPT clustered with the *Prevotella*-dominated enterotype, and samples with a relatively high MPT clustered with the *Bacteroides*-dominated enterotype (Fig. 4g). The low predicted temperature of the *Prevotella*-dominated enterotype was consistent with the lower predicted OGT of *Prevotella* species (26.48 ± 3.27°C) compared with that of *Bacteroides* species (34.46 ± 2.42°C) (Supplementary Table S8). Finally, we investigated whether gender affected the distribution of samples on the LEA map (Fig. 4h). The results revealed a larger number of male samples were located around the *Prevotella*-dominated enterotype area than female samples, which is consistent with the result that the *Prevotella*-dominated cluster had a larger proportion of male samples in the study that produced this dataset.^25^

## 4. Discussion

We developed the Metagenomic Thermometer as a means of predicting environmental temperature based on *F*_*IVYWREL*_ value for coding sequences of metagenomic sequence data. The correlation between *F*_*IVYWREL*_ and OGT has been confirmed only in prokaryotes, so it is unclear how eukaryotic and viral sequences affect to the temperature prediction. However, previous studies have indicated that metagenomic sequences generally contain relatively small proportion of eukaryotic and viral sequences.^50,51^ Furthermore, in the metagenomic sequences used in this study, the proportion of sequences classified as eukaryotes and viruses was also limited (Supplementary Fig. S4). Although the Metagenomic Thermometer predicted temperature well for stable environments, three environmental conditions were identified that adversely affect prediction accuracy. The first is environments with an influx of microorganisms from the outside. Sewage and plant water samples shows elevated MPT, which is partially explained by the presence of mesophilic bacteria derived from human gut that are adapted to human body temperature (Fig. 1c and Supplementary Fig. S1). The second is environments with low temperature. In soil, stream, and ocean, low-temperature samples tended to yield higher MPTs than actual temperatures (Fig. 1b–d). This may be because cold adaptation lacks specific patterns of amino-acid composition changes, in contrast to high-temperature adaptation.^52^ In fact, the OGT of psychrophilic microbes is difficult to predict using amino-acid composition.^11,53^ The third is environments with large temperature changes. Figure 1e shows that the MPT of ocean samples changes with a delay of about 2 months from the actual temperature changes. This result suggests that it takes a long time for microbial communities to respond to changes in environmental temperature. This is an important finding obtained by using the Metagenomic Thermometer, which is difficult to decipher from the phylogenetic composition.

Application of the Metagenomic Thermometer to human gut metagenomic data results in MPT close to the normal human body temperature. In addition, MPT was consistent with the well-known fact that the body temperature of human females is higher than that of males. In general, measuring human intestinal temperature requires physical stress or special instruments such as electronic capsules.^54^ Our results demonstrate the potential use of Metagenomic Thermometers to measure intestinal temperature in humans or livestock. The result of LEA mapping shows that males have more *Prevotella* enterotypes than females. The predominance of *Prevotella* in the male gut has been reported in several studies.^55–57^ Although there is a study suggesting a sex hormone relationship to *Prevotella* abundance,^58^ exact cause of *Prevotella* predominance in the male gut remains unclear.^55–57^ Our results suggest that low deep body temperature may be a factor in causing *Prevotella* predominance in the male gut. Furthermore, the results suggest that body temperature determines the microorganisms that can establish themselves in the gut. The Metagenomic Thermometer enables the design of community composition of LBPs (Live Biotherapeutic Products) or genomic sequences of probiotic bacteria that are personally optimized for individual deep body temperature.

The community composition analysis of the artificial hot water canal revealed that the environmental temperature and the OGT of the existing microorganisms are somewhat consistent. However, microbial taxa with a wide range of OGT coexist at each sampling point in the canal (Fig. 3b). The Metagenomic Thermometer is feasible not simply because the environment is occupied by microbes with appropriate OGTs, but because the amino acid fraction is balanced as a whole community. Microbial community composition is affected by characteristics other than temperature, such as pH^19^ and chemical composition.^28,59^ Our analysis of hot spring metagenomic data also showed that pH affected microbial communities as strongly as temperature (Supplementary Fig. S5a and b). However, pH did not impart any bias to the results of the Metagenomic Thermometer (Supplementary Fig. S5c). This is thought to be due to differences in the characteristics of environmental factors. Because temperature cannot be regulated across the bacterial cell membrane, all intracellular proteins are affected by the external temperature. On the other hand, acidophiles cope with acidity by keeping the intracellular pH close to neutral,^60^ so it is not important that whole intracellular protein acquires acid resistance. The results of hot spring metagenomic analysis show that the Metagenomic Thermometer was not subject to changes in taxonomic composition. The understanding of community assembly mechanisms has long been debated, yet a unified understanding has not been reached.^61^ One study revealed that niche-specific deterministic community assembly processes have a greater effect on gene function than on overall taxonomy.^62^ Our study underscores the importance of seeing microbial communities not only as a collection of species but also as a collection of more fundamental biomolecules such as nucleotides or amino acids.

## Supplementary Material

dsad024_suppl_Supplementary_Figures_S1

dsad024_suppl_Supplementary_Figures_S2

dsad024_suppl_Supplementary_Figures_S3

dsad024_suppl_Supplementary_Figures_S4

dsad024_suppl_Supplementary_Figures_S5

dsad024_suppl_Supplementary_Tables_S1-S8

dsad024_suppl_Supplementary_Data

## Data Availability

Metagenomic sequencing reads were deposited in the DNA Data Bank of Japan with accession number DRA012733. All other data are available in the Supplementary material. A STORMS (Strengthening The Organizing and Reporting of Microbiome Studies) checklist and raw data for taxonomic compositions are available at https://zenodo.org/record/7247214#.Y1eGjezP1qt. Metagenomic Thermometer is an open-source software available at https://github.com/kuroppy/meta-thermo as a downloadable Python package. Metagenomic Thermometer web application we developed can be freely accessed at http://metathermo.jp/.
