# Supplementary figures and images for: Metagenomic Thermometer
Source: DNA Res. 2023 Nov 6;30(6):dsad024. doi: 10.1093/dnares/dsad024 (PMC10660216; doi:10.1093/dnares/dsad024)

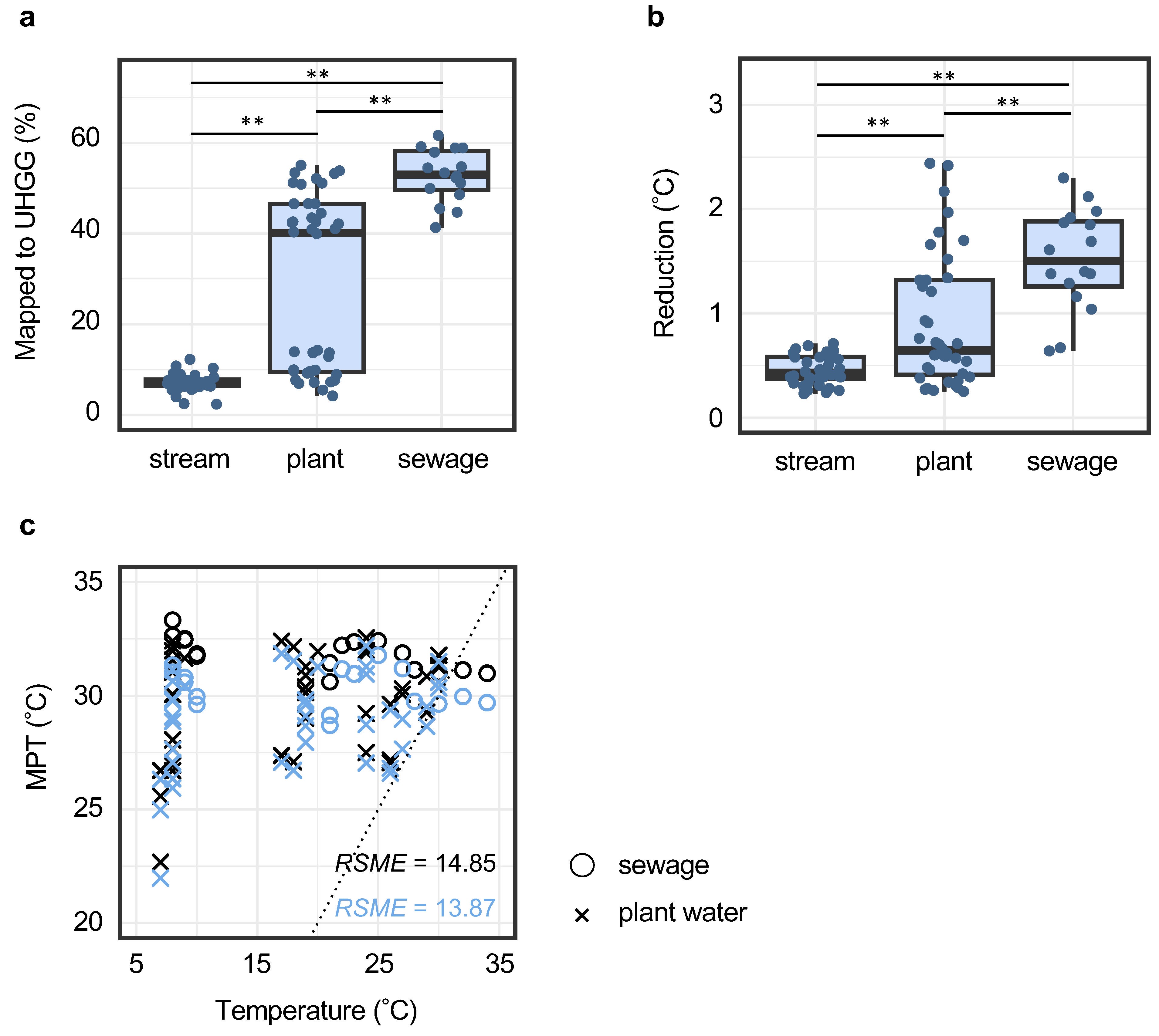

Supplement: dsad024_suppl_Supplementary_Figures_S1 [file dsad024_suppl_supplementary_figures_s1.jpeg]

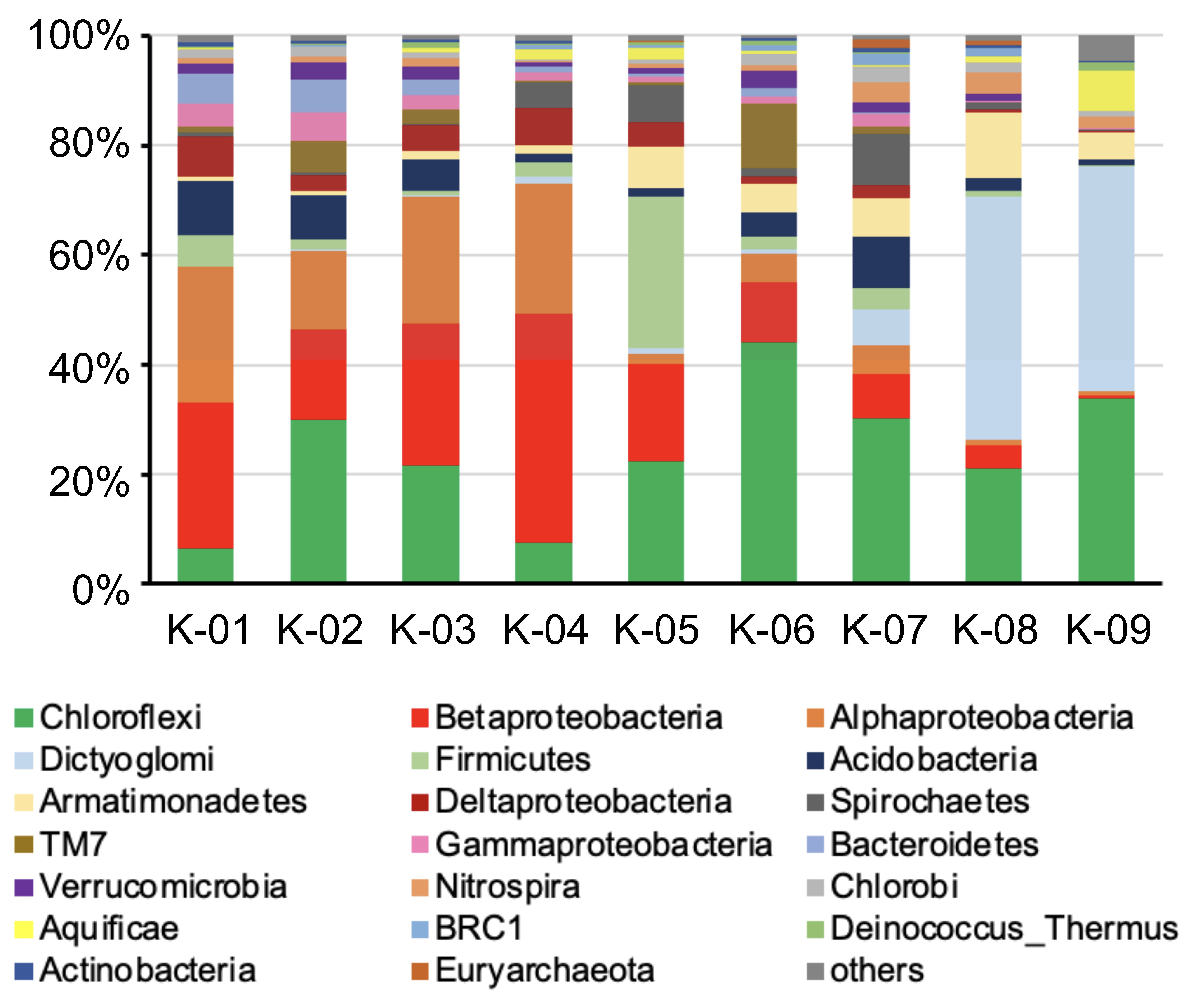

Supplement: dsad024_suppl_Supplementary_Figures_S2 [file dsad024_suppl_supplementary_figures_s2.jpeg]

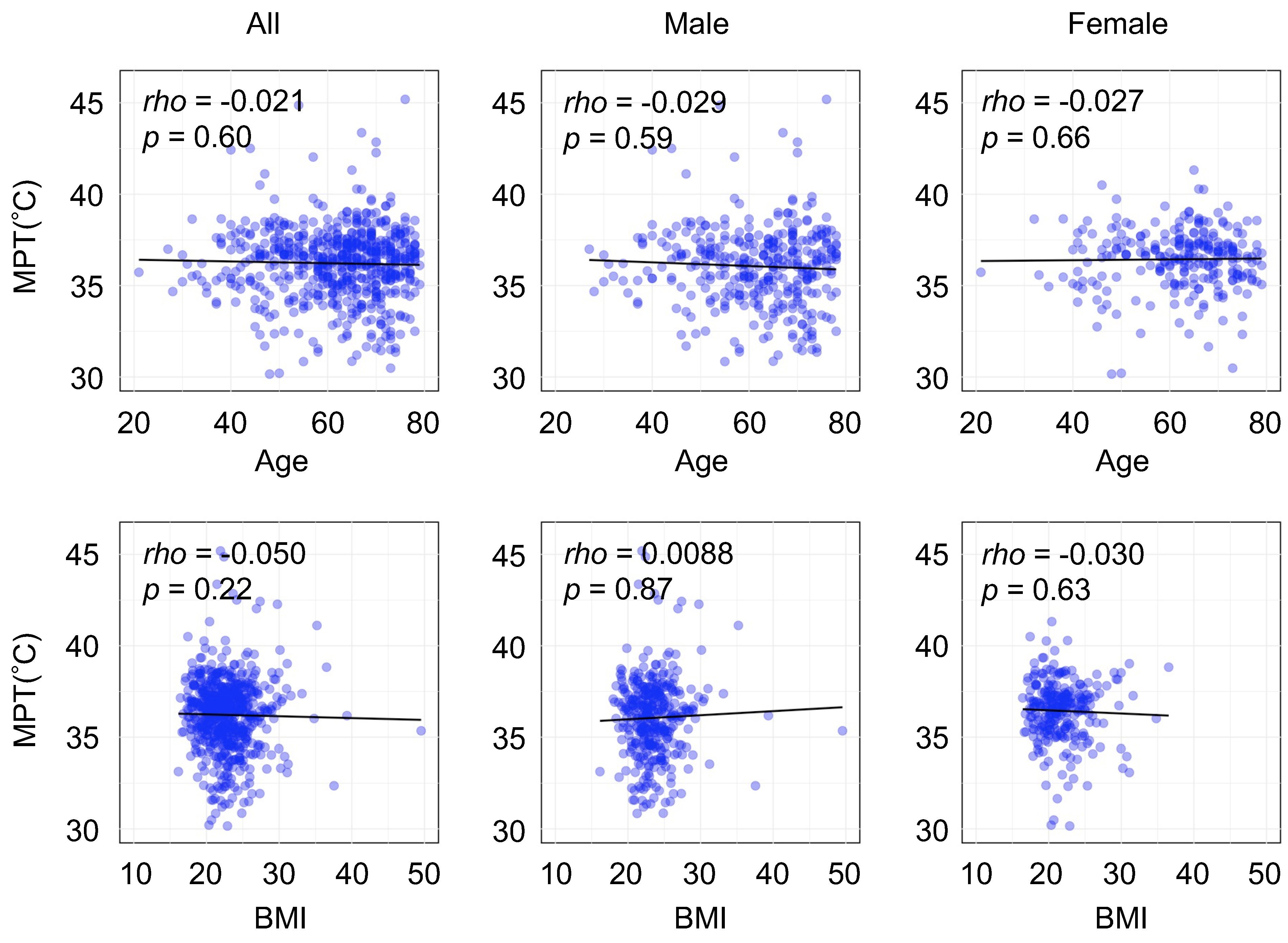

Supplement: dsad024_suppl_Supplementary_Figures_S3 [file dsad024_suppl_supplementary_figures_s3.jpeg]

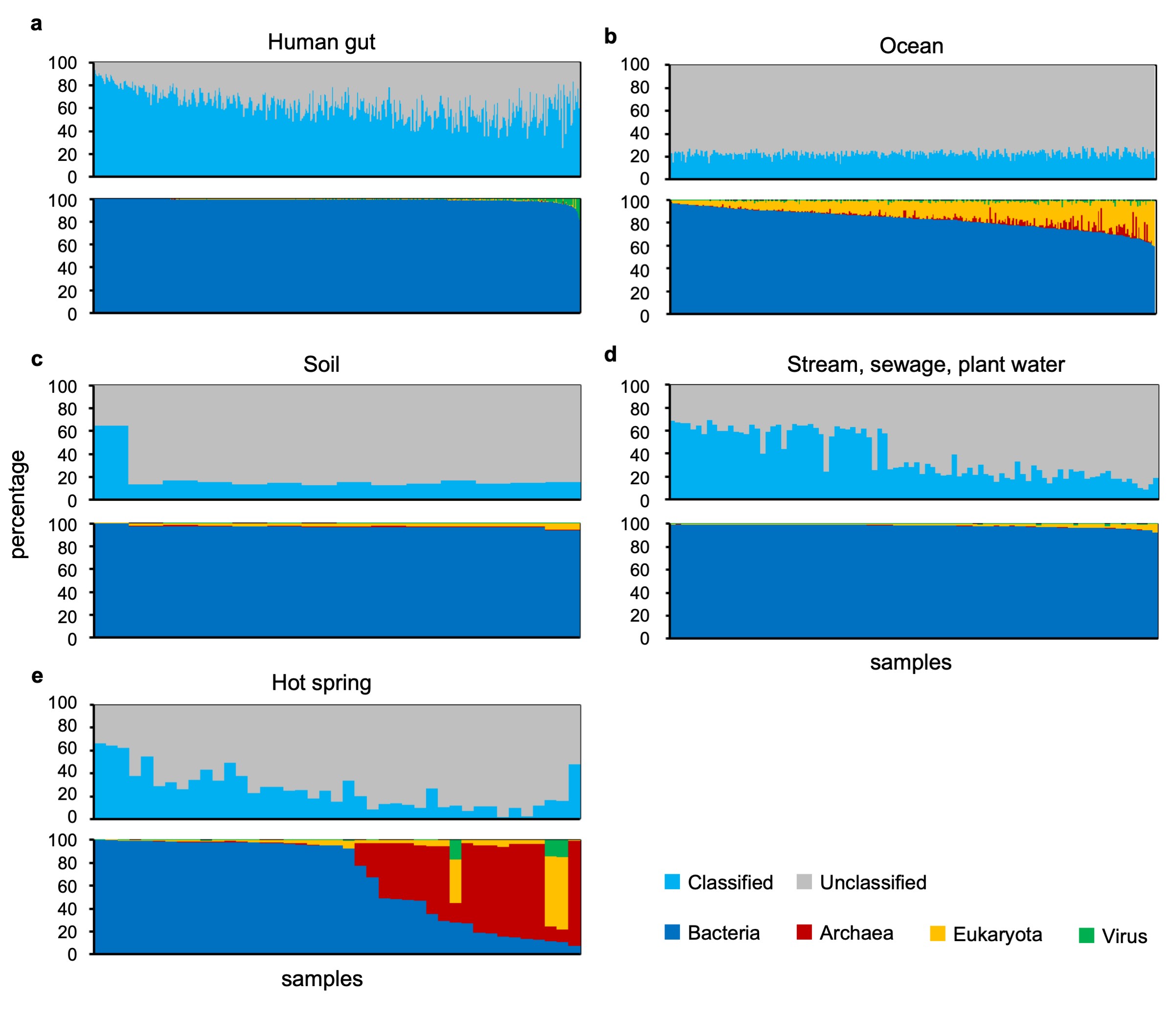

Supplement: dsad024_suppl_Supplementary_Figures_S4 [file dsad024_suppl_supplementary_figures_s4.jpeg]

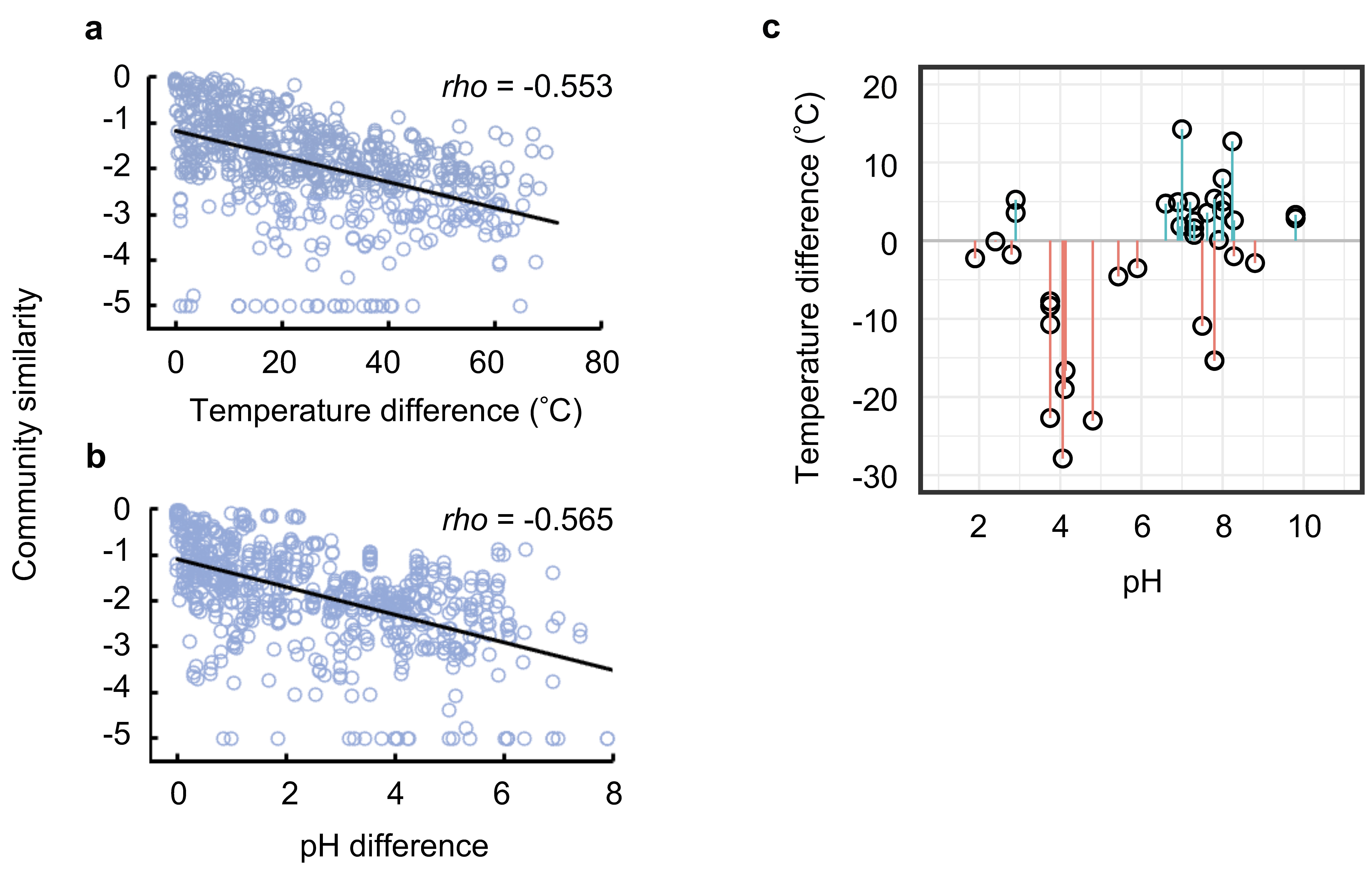

Supplement: dsad024_suppl_Supplementary_Figures_S5 [file dsad024_suppl_supplementary_figures_s5.jpeg]
