# Supplementary material for: Metagenomic Thermometer
Source: DNA Res. 2023 Nov 6;30(6):dsad024. doi: 10.1093/dnares/dsad024 (PMC10660216; doi:10.1093/dnares/dsad024)
Supplement: dsad024_suppl_Supplementary_Data [file dsad024_suppl_supplementary_data.docx]

**Supplementary Figure Legends**

**Fig. S1 Removing sequences of human gut microbe**

(a) The boxplots represent the proportion of metagenomic sequences mapped to the UHGG. Results are shown separately for stream, plant water, and sewage. ** indicates p < 0.01 for a two-tailed Welch's t-test. (b) The boxplots represent the reduction of MPT due to removal of reads mapped to UHGG. ** indicates p < 0.01 for a two-tailed Welch's t-test. (c) Scatter plots shows the predicted temperatures for data before and after filtering the human gut microbiota sequences shown in black and blue, respectively. The points represent data from the plant water and sewage as indicated in the legend. The *RSME* between the predicted and measured temperatures is calculated for each of the before and after filtering data.

**Fig, S2 Community structure examined by 16S amplicon sequencing**

The 20 most abundant phyla or class (for Proteobacteria) in all samples and the remaining phyla correspond to the colors of the legend. Labels at the bottom represent the samples.

**Fig. S3 Correlation analysis between predicted temperature and individual characteristics**

Correlation between MPT and age (upper) and body mass index (BMI; lower). The left-most graphs present the data for all samples, whereas the middle and right graphs present data for samples pertaining to males and females, respectively. Each black line indicates the linear regression line. Spearman’s correlation coefficients and p-values are shown in each graph.

**Fig. S4 Domain composition of metagenomic sequences**

Bar charts show domain composition of each metagenomic sequences from human gut (a), ocean (b), soil (c), stream, sewage, and plant water (d), and hot spring (e). Upper bar charts show the proportion of reads classified into any domain by Kraken2, while lower ones show the domain ratios of classified reads. Each bar corresponds to one sample and is arranged from left to right based on the proportion of Bacteria. The samples in the upper and lower graphs are arranged in the same order.

**Fig. S5 Effects of pH and temperature on microbial communities**

Community similarity was calculated for all pairs of 41 hot spring metagenomes. Pairwise community similarity decreases according to difference of temperature (a) or pH (b) between samples increases (p < 2.2 × 10–16). ‘Community similarity’ was calculated as 1 minus the calculated Bray-Curtis dissimilarity and taken as the common logarithm. (c) Relationship between pH and temperature prediction difference. Temperature difference was calculated by subtracting the actual temperature from the MPT. The magnitude of the difference is shown in different colors corresponding to positive and negative.
